# Primary ovarian parasitic diseases mimicking ovarian cancer in the reproductive system: diagnostic challenges and surgical challenges (a case report)

**DOI:** 10.11604/pamj.2025.52.35.48948

**Published:** 2025-09-23

**Authors:** Fang Zhang, Qinwei Zhang, Gun Chen, Weili Yang, Jianbin Li

**Affiliations:** 1 Gynecology Department, the Affiliated People's Hospital of Ningbo University, Ningbo, 315000, Zhejiang, China

**Keywords:** Ovarian neoplasms, parasitic diseases, echinococcosis, pelvic abscess, case report

## Abstract

Primary ovarian parasitic disease is rare and can masquerade as advanced ovarian carcinoma; we report the first detailed case in a post-menopausal woman that underscores this diagnostic trap. A 51-year-old woman with chronic raw-seafood ingestion presented with bilateral adnexal masses (7cm left, 5cm right), low-grade fever, leukocytosis (13.2 × 10^9^/L), markedly elevated C-reactive protein (135mg/L) and CA-125 (69.5U/mL), and imaging suggesting ovarian malignancy with ascites. Intra-operatively, a “frozen pelvis” with dense adhesions, multiple abscesses and omental cake was found; frozen section excluded neoplasia, leading to total hysterectomy, bilateral salpingo-oophorectomy, appendectomy and abscess debridement guided by infectious-disease principles. Histopathology revealed chronic suppurative granulomatous inflammation with parasitic remnants consistent with echinococcal disease, and the patient remains disease-free at 5 years. Clinicians should include parasitic infection in the differential of complex pelvic masses in patients with raw-food exposure; complete excision adhering to anti-infective surgical protocols prevents misdiagnosis as malignancy and avoids unnecessary oncologic overtreatment.

## Introduction

Primary ovarian echinococcosis is a rare differential diagnosis in gynaecologic oncology, with a pooled incidence of 0.2-2.25% among pelvic parasitic diseases and ≤2% of all abdominal hydatidosis [1,2]. Its clinical and radiological overlap with malignant ovarian neoplasms frequently results in misclassification and unnecessary radical surgery. Nonspecific biomarkers-elevated CA-125, leukocytosis, and raised C-reactive protein are common to both entities and therefore unreliable for discrimination [3]. The pathogenesis of ovarian echinococcosis remains incompletely understood. In contrast to hepatic or pulmonary disease, which account for 70-90% of cases [4], genital-tract involvement typically follows rupture of adjacent visceral cysts or haematogenous dissemination of *Echinococcus granulosus* oncospheres. Humans acquire infection by ingesting embryonated eggs in contaminated food or water; consumption of raw seafood has been identified as a key risk factor [5]. Whereas hepatic echinococcosis exhibits well-defined imaging hallmarks such as the “water-lily sign”, the ovary lacks pathognomonic radiological features. Consequently, ultrasonography, Computed Tomography scan (CT) and Magnetic Resonance Imaging (MRI) often assign these lesions to O-RADS 4, a category denoting intermediate malignancy risk [6].

In non-endemic regions, the misdiagnosis rate exceeds 85%. A recent retrospective series reported that 100% of ovarian hydatid cysts (n=7) were initially managed as primary ovarian tumours [1]. This diagnostic failure is attributable to (i) symptom mimicry chronic pelvic pain, adnexal masses and secondary abscess formation imitate advanced ovarian carcinoma; (ii) limitations of frozen-section analysis, which typically reveals only nonspecific inflammation and fails to identify parasitic elements; and (iii) incomplete exposure histories, with >80% of misdiagnosed patients lacking documentation of residence in endemic areas or raw-food consumption [1,3].

Optimal management remains controversial. Complete cystectomy is curative in uncomplicated cases, yet abscess formation or a “frozen pelvis” mandates extended resection according to pelvic-sepsis protocols rather than oncological guidelines, a strategy essential to prevent anaphylaxis or disseminated disease [7]. Emerging serological assays such as ELISA targeting antigen B (EgAgB) and diffusion-weighted MRI show promise, but prospective validation in ovarian disease is still required.

## Patient and observation

**Figure 1:**
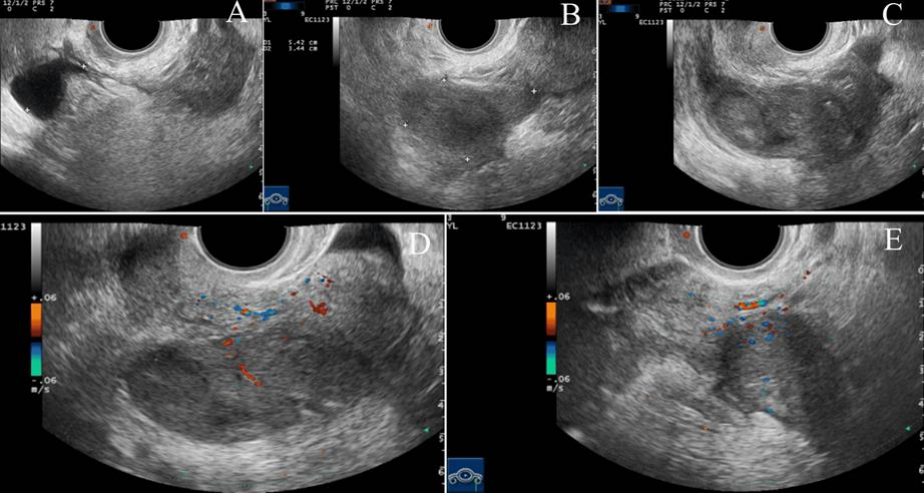
transvaginal ultrasound: A) sagittal: 29mm free fluid (*); B) right: 54×34mm hypoechoic mass, angular margins, flow; C) left: 74×34mm heterogeneous lesion, 3-5mm foci; D) left: moderate flow, RI 0.42; E) right: marked peri-/central flow

**Patient information:** in April 2019, a 51-year-old postmenopausal woman (G1P1) presented with incidentally detected bilateral adnexal masses on routine transvaginal ultrasound, revealing a 74×34mm irregular hypoechoic lesion with heterogeneous echotexture, fused morphology, restricted mobility, and 3-5mm hyperechoic foci in the left adnexa (CDFI vascularity; RI 0.42), alongside a 54×34mm hypoechoic mass with angular margins in the right adnexa (CDFI positive), accompanied by 29-mm fluid in the pouch of Douglas (Figure 1); she denied abdominal pain, vaginal bleeding, nausea, or vomiting, had undergone uncomplicated cesarean section 27 years prior, reported lifelong coastal residence in eastern China with habitual raw seafood consumption (≥3 times/week including marinated crustaceans), and had no endemic parasitic exposure history.

**Clinical findings:** on admission, the patient exhibited low-grade fever (37.7°C) with stable hemodynamic parameters; gynecological examination revealed a nontender uterus and bilateral adnexal masses-a 7-cm well-defined, fixed mass in the left adnexal region demonstrating restricted mobility, and a 5-cm well-demarcated, moderately mobile mass in the right adnexa without cervical motion tenderness or abnormal discharge.

**Timeline of the current episode:** the patient was admitted on April 29^th^, 2019, presenting with persistent low-grade fever (peak 38.0°C) without abdominal symptoms; empirical cefmetazole therapy (2g IV q12h) achieved normalization of leukocytosis and body temperature within 6 days, followed by exploratory laparotomy on May 9^th^, 2019, which revealed extensive pelvic pathology necessitating definitive surgery, with subsequent discharge on May 22^nd^, 2019, and annual surveillance confirming disease-free status through 6-year follow-up without recurrence.

**Figure 2:**
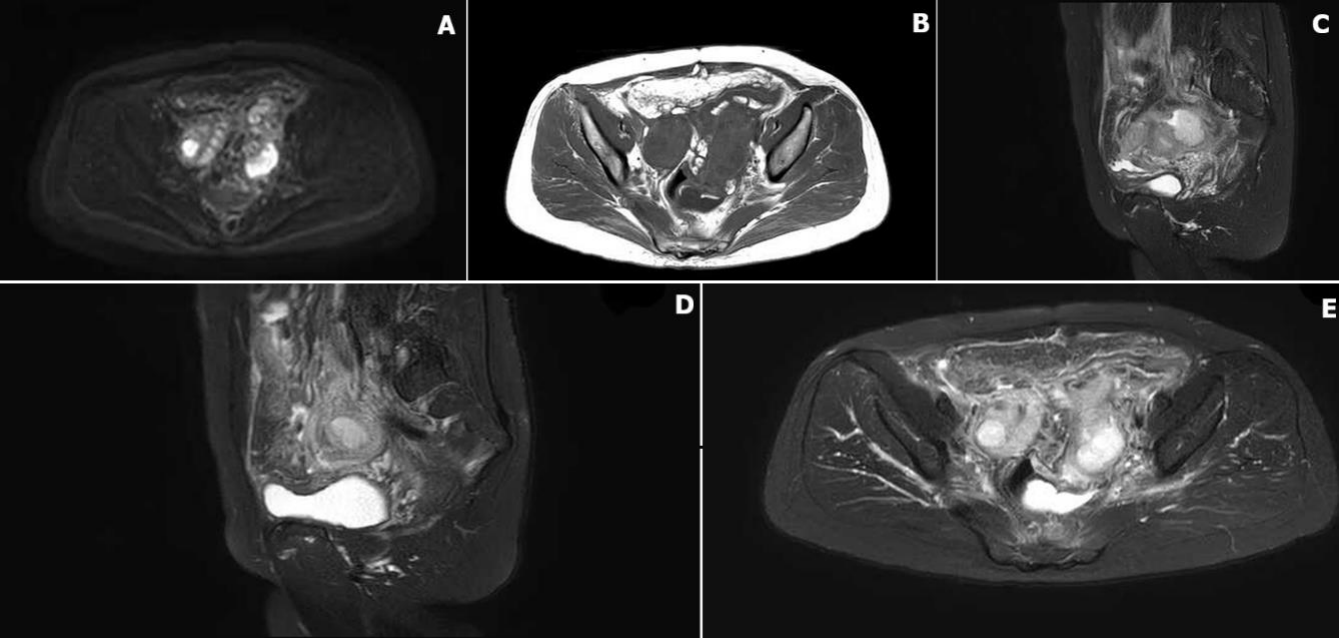
pelvic MRI: A) DWI: restricted diffusion, purulent cysts; B) axial T1WI: isointense to myometrium; C,D) sagittal T2WI: hyperintense masses, cystic foci; E) axial T2WI: complex cystic-solid lesions, stranding

**Figure 3:**
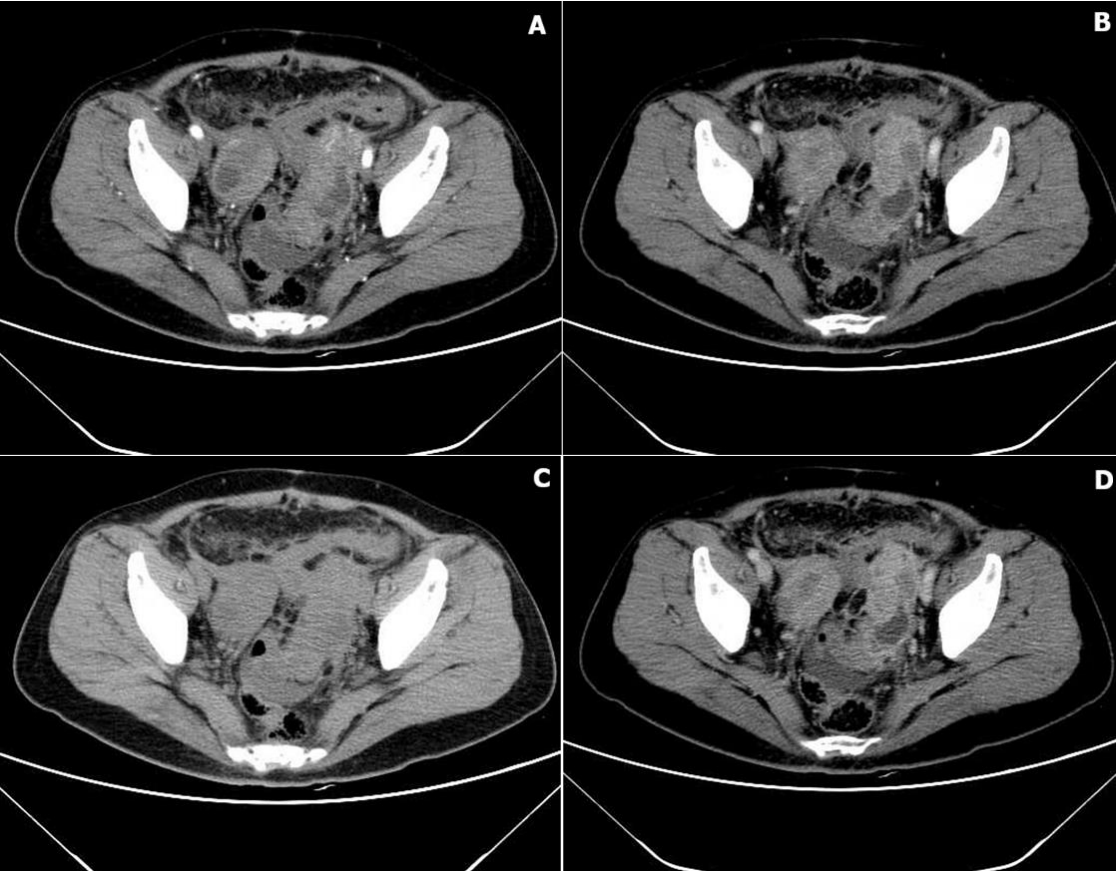
contrast CT: A) arterial: mild heterogeneous enhancement; B) venous: centripetal enhancement; C) unenhanced: mixed cystic-solid, lobulated, stranding; D) delayed: solid persists; cystic hypodense (*)

**Diagnostic assessment:** the physical examination revealed the following vital signs: Laboratory investigations revealed elevated tumour markers (TSGF 136.8 U/mL, CA125 69.5 U/mL), leukocytosis (13.2×10^9^/L) with neutrophilia (83.8tivity (4.85×10^4^ copies/mL); pelvic MRI demonstrated bilateral adnexal masses with intermediate T2 signal and minimal pelvic fluid (Figure 2), while contrast-enhanced abdominal CT showed ill-defined soft tissue masses in both ovarian regions with pelvic fat stranding and multiple subcentimeter nodules, mildly enlarged retroperitoneal/mesenteric lymph nodes, a hypodense lesion in liver segment V (arterial phase), and a right renal cyst (Figure 3); gastrointestinal endoscopy identified chronic superficial gastritis and a rectal protrusion suggestive of extrinsic compression versus infiltration.

**Diagnosis:** primary ovarian echinococcosis with granulomatous inflammation (pathologically confirmed), bilateral tubo-ovarian abscesses (secondary to parasitic infection), chronic endometritis with focal granulomatous changes, chronic cervicitis, chronic catarrhal appendicitis, cholecystic polyp (incidental finding), chronic superficial gastritis, simple renal cyst, right kidney.

**Figure 4:**
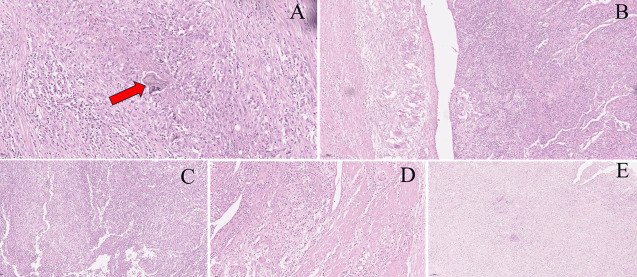
histology: A) parasite in granuloma (×200) (red arrow); B) epithelioid granulomas, giant cells (×100); C) cleft necrosis, foamy histiocytes (×100); D) eosinophils with neutrophils, macrophages (×200); E) adjacent peritoneal fibrous tissue exhibiting diffuse aggregates of epithelioid histiocytes and numerous multinucleated giant cells forming a florid granulomatous reaction (H&E, ×100; scale bar = 200µm)

**Therapeutic interventions:** the patient underwent exploratory laparotomy revealing extensive pelvic pathology: cake-like omental thickening with dense adhesions to multiple colonic and small bowel segments, a severely edematous appendix (6×4×4cm), uterus encased by inflammatory rind, bilateral adnexa matted into 6×5cm masses with extension to the rectal surface, rectal serosal nodular necrosis, and congested bowel loops; intraoperative frozen section of omentum and uterus/adnexa demonstrated chronic inflammation with epithelioid reaction and chronic suppurative salpingitis without malignancy, prompting surgical management per pelvic abscess protocol via total hysterectomy-bilateral salpingo-oophorectomy (TH-BSO), appendectomy, omentectomy, resection of rectovaginal nodule, peritoneal biopsies, and rectal/small bowel mesentery sampling; definitive histopathology confirmed chronic endometritis with granulomatous foci, bilateral adnexal suppurative inflammation with parasitic remnants, appendiceal catarrhal inflammation, and fibroinflammatory changes in all resected specimens (Figure 4); postoperative evaluation included negative tuberculin testing and brain MRI ruling out metastasis, culminating in uncomplicated recovery and discharge on postoperative day 10.

**Follow-up and outcomes:** through a structured 6-year surveillance protocol involving annual physical examinations, serum CA125 monitoring, and pelvic imaging, the patient maintained disease-free status with normalized inflammatory markers (CRP <5 mg/L, ESR <20mm/h), absence of adnexal recurrence on serial ultrasounds, stable CA125 levels (range 12-18 U/mL), and no evidence of parasitic reactivation or distant dissemination, confirming sustained remission without adjunctive therapy at the final follow-up in May 2025.

**Patient perspective:** the patient was delighted with the quality of care.

**Informed consent:** written informed consent was obtained from the patient for the publication of this case report.

## Discussion

Primary ovarian echinococcosis is a rare presentation of cystic hydatid disease, representing only 0.2-1% of all intra-abdominal cases [8]. In non-endemic regions, more than 85% are misdiagnosed because the classic hepatic focus is absent, tumour markers such as CA-125 may be only mildly elevated, and imaging often fulfils criteria for ovarian malignancy (O-RADS 4) [6]. The present patient exhibited the so-called “frozen pelvis” with bilateral adnexal masses, a pattern that has been mistaken for advanced carcinoma in 57-100% of published series [6]. These data underscore the necessity of including parasitic aetiology in the differential diagnosis of any pelvic mass, particularly when epidemiological clues, such as habitual raw seafood consumption observed here and now.

The route by which *Echinococcus granulosus* reaches the ovary remains speculative: haematogenous seeding and retrograde lymphatic spread are the most widely accepted hypotheses [9]. Two unusual immunological features were noted. First, overt eosinophilia was absent (0.1%) despite marked systemic inflammation (CRP 135 mg/L). Experimental studies suggest that parasite-derived extracellular vesicles containing miRNA-277 suppress eosinophil chemotaxis, facilitating immune evasion [8]. Second, concomitant Ureaplasma urealyticum infection (4.85×10^4^ copies mL^-1^) was detected. Ureaplasma activates TLR4/NF-κB signalling and may amplify granulomatous inflammation while simultaneously masking the underlying parasite. The pronounced neutrophilia (83.8%) further implies activation of the NLRP3 inflammasome-possibly triggered by parasite heat-shock protein 20-as recently reported in chronic echinococcosis.

Current guidelines recommend simple cystectomy for uncomplicated hydatid cysts, yet abscess formation and dense pelvic adhesions necessitated extended surgery (total hysterectomy with bilateral salpingo-oophorectomy, omentectomy and appendicectomy). Radical resection achieves complete excision and avoids the 40% recurrence risk associated with incomplete debulking; in the present case, five-year follow-up has remained disease-free [8]. Intra-operative frozen section ruled out malignancy but did not identify parasitic elements, most likely because of sampling error within dense granulomatous tissue. Whenever necrotising granulomas are encountered, targeted stains such as calcofluor white should be requested. Empiric cefmetazole transiently suppressed fever and leucocytosis, presumably by controlling the Ureaplasma co-pathogen, but inadvertently delayed the correct diagnosis. This observation suggests that antibiotic trials for suspected pelvic inflammatory disease in endemic areas should include empirical antiparasitic coverage.

The patient's dietary history implicates *E. granulosus* genotype G1, the Asian marine strain increasingly recognised in coastal outbreaks [8]. We therefore advocate the routine implementation of preoperative serum EgAgB ELISA (demonstrating 94-98% sensitivity for pelvic echinococcosis) to mitigate diagnostic delays, with concurrent adoption of diffusion-weighted MRI biomarkers, specifically rim-restricted diffusion (ADC<1.2×10^-3^ mm^2^/s) and T2-hypointense cyst walls, as key imaging discriminators between hydatid cysts and neoplastic lesions.

Absence of parasite genotyping and lack of serial serology limit the strength of our conclusions. Emerging tools nevertheless offer promise: CRISPR-based SHERLOCK assays targeting Echinococcus cell-free DNA in peritoneal fluid may permit rapid intra-operative confirmation, and mitochondrial uncouplers such as BAM15, shown to reduce Toxoplasma cyst burden by 89%, could be investigated for hydatid disease.

## Conclusion

Primary ovarian echinococcosis must be considered whenever an adnexal mass is accompanied by atypical tumour markers or epidemiological risk factors. Early EgAgB ELISA, multidisciplinary surgical planning, and a low threshold for parasitological consultation can shift management from radical resection to conservative cystectomy, thereby reducing surgical morbidity while ensuring cure.
